# 3D Printed Integrated Multi-Layer Microfluidic Chips for Ultra-High Volumetric Throughput Nanoliposome Preparation

**DOI:** 10.3389/fbioe.2021.773705

**Published:** 2021-10-11

**Authors:** Han Shan, Qibo Lin, Danfeng Wang, Xin Sun, Biao Quan, Xiang Chen, Zeyu Chen

**Affiliations:** ^1^ Department of Dermatology, Xiangya Hospital, Central South University, Changsha, China; ^2^ School of Mechanical and Electrical Engineering, Central South University, Changsha, China

**Keywords:** liposome, microfluidic, 3D printing, high volumetric throughput, multi-layer layout

## Abstract

Although microfluidic approaches for liposomes preparation have been developed, fabricating microfluidic devices remains expensive and time-consuming. Also, owing to the traditional layout of microchannels, the volumetric throughput of microfluidics has been greatly limited. Herein an ultra-high volumetric throughput nanoliposome preparation method using 3D printed microfluidic chips is presented. A high-resolution projection micro stereolithography (PμSL) 3D printer is applied to produce microfluidic chips with critical dimensions of 400 µm. The microchannels of the microfluidic chip adopt a three-layer layout, achieving the total flow rate (TFR) up to 474 ml min^−1^, which is remarkably higher than those in the reported literature. The liposome size can be as small as 80 nm. The state of flows in microchannels and the effect of turbulence on liposome formation are explored. The experimental results demonstrate that the 3D printed integrated microfluidic chip enables ultra-high volumetric throughput nanoliposome preparation and can control size efficiently, which has great potential in targeting drug delivery systems.

## Introduction

Liposomes are spherical vesicles consisting of one or more phospholipid bilayers, can remarkably improve the permeability of encapsulated cargo within the target tissue, and have been widely employed for biomedical applications, such as nanomedicines and contrast agent (Ringgaard et al., 2020; Che et al., 2020; Kim et al., 2019; Liu et al., 2017; Chen et al., 2017). Owing to their ideal size and excellent biocompatibility, liposomes are the promising carrier for a variety of agents, including drugs, small interference RNA (siRNA), plasmid DNA, etc. There are many methods to prepare liposomes (Pattni et al., 2015; Shah et al., 2020; Akbarzadeh et al., 2013), such as thin film hydration, reverse phase evaporation, and ether or ethanol injection.

The micro hydrodynamic focusing (MHF) method, first described by Jahn et al. (Jahn et al., 2004), is a novel method to form liposomes *via* microfluidic devices, aiming to address the issues of poor reproducibility. Due to the sub-millimeter scale of the microchannels, the flows that have low Reynolds Number (*Re*) achieve an ideal diffusive mixing effect, contributing to the generation of small size liposomes (from 50 to 150 nm). Compared with other macroscale methods, the MHF method can precisely control the liposome size distribution. While microfluidic approaches of preparing micro or nanoparticles have been developed (Jahn et al., 2004; Jahn et al., 2010; Hood et al., 2014; Shi et al., 2020; Deng et al., 2016; Carugo et al., 2016; Song et al., 2018; Liu et al., 2021; Park et al., 2010; Liu et al., 2015), the fabrication of microfluidic devices remains high cost and time-consuming. A wide variety of materials have been explored to fabricate microfluidic chips, such as polymethyl methacrylate (PMMA) (Sathish et al., 2019), polydimethylsiloxane (PDMS) (Rahman et al., 2020), glass (Liu et al., 2015), silicon (Chung et al., 2008), and even papers (Ainla et al., 2017). Typically, the main structure of the microfluidic chip contains two parts, that is, the microchannel layer and substrate. The manufacturing process of microfluidic chips may involve photolithography, deep reactive-ion etching (DRIE), casting and bonding procedures, which not only requires special equipment, but also a cleaning room environment. Moreover, the inlets of the microfluidic chip are required to bond with the extra devices, e.g., poly-ether-ether-ketone (PEEK) connectors, for ensuring a stable inflow of aqueous samples.

Several methods have been developed for fabricating polymer devices rapidly, such as additive manufacturing, laser cutting and roll-to-roll (R2R) hot embossing (Xiang et al., 2019; Wang et al., 2016; Zhang et al., 2016; Xiang et al., 2019). Additive manufacturing, also called 3D printing, is a promising and cost-effective technique, which has been employed in the fabrication of microfluidic chips (Balakrishnan et al., 2021; Su et al., 2020; Li et al., 2020; Xiang et al., 2018). 3D printing methods have features of rapid prototyping, flexible design, and low cost, therefore the 3D printed microfluidic devices have received tremendous attention in the field of nanoparticles preparation (Bishop et al., 2015; Chen et al., 2019; Tiboni et al., 2020). Chen et al. proposed the strategy that liposomes were synthetized by using a 3D printed high throughput microfluidic device (Chen et al., 2019). By optimizing structures of 3D printed microfluidic chips, they achieved total volumetric flow rates as high as 30 ml min^−1^. However, limited by the resolution of 3D printer and resin, the 3D printer needs to be carefully handled during printing, which hinders the mass production of microfluidic chips.

Nowadays, it remains challenging to realize a high volumetric throughput liposome preparation *via* microfluidic devices. The larger the volumetric throughput, the higher the injection pressure, which brings the risk of structure destruction. On the other hand, a higher volumetric throughput commonly brings the transition from laminar to turbulence, which may limit the formation of smaller liposomes (Jahn et al., 2010). Here, we report an ultra-high volumetric throughput nanoliposome preparation method by using 3D printed microfluidic chips. A projection micro stereolithography (PμSL) 3D printer (with 10 μm X-Y plane resolution) is introduced to ensure the rapid prototyping of microfluidic chips. For achieving a high throughput liposome production, the microchannels of the microfluidic chip adopt a three-layer layout, contributing to total flow rate (TFR) values up to 474 ml min^−1^, to our best knowledge, it is the highest TFR value in the reported liposome formation microfluidic chips. The liposome size distribution is evaluated with a variety of TFRs and flow rate ratios (FRRs). The state of flows in microchannels, laminar or turbulence, is explored with the increasing FRRs and TFRs, and the liposome formation mechanism under turbulence conditions is also explained. The experimental results exhibit that the 3D printed microfluidic chip features ultra-high volumetric throughput and can prepare liposomal nanoplatforms with controlled size rapidly, which has great potential in targeting drug delivery systems.

## Materials and Methods

### Fabrication of Microfluidic Chips

**FIGURE 1:**
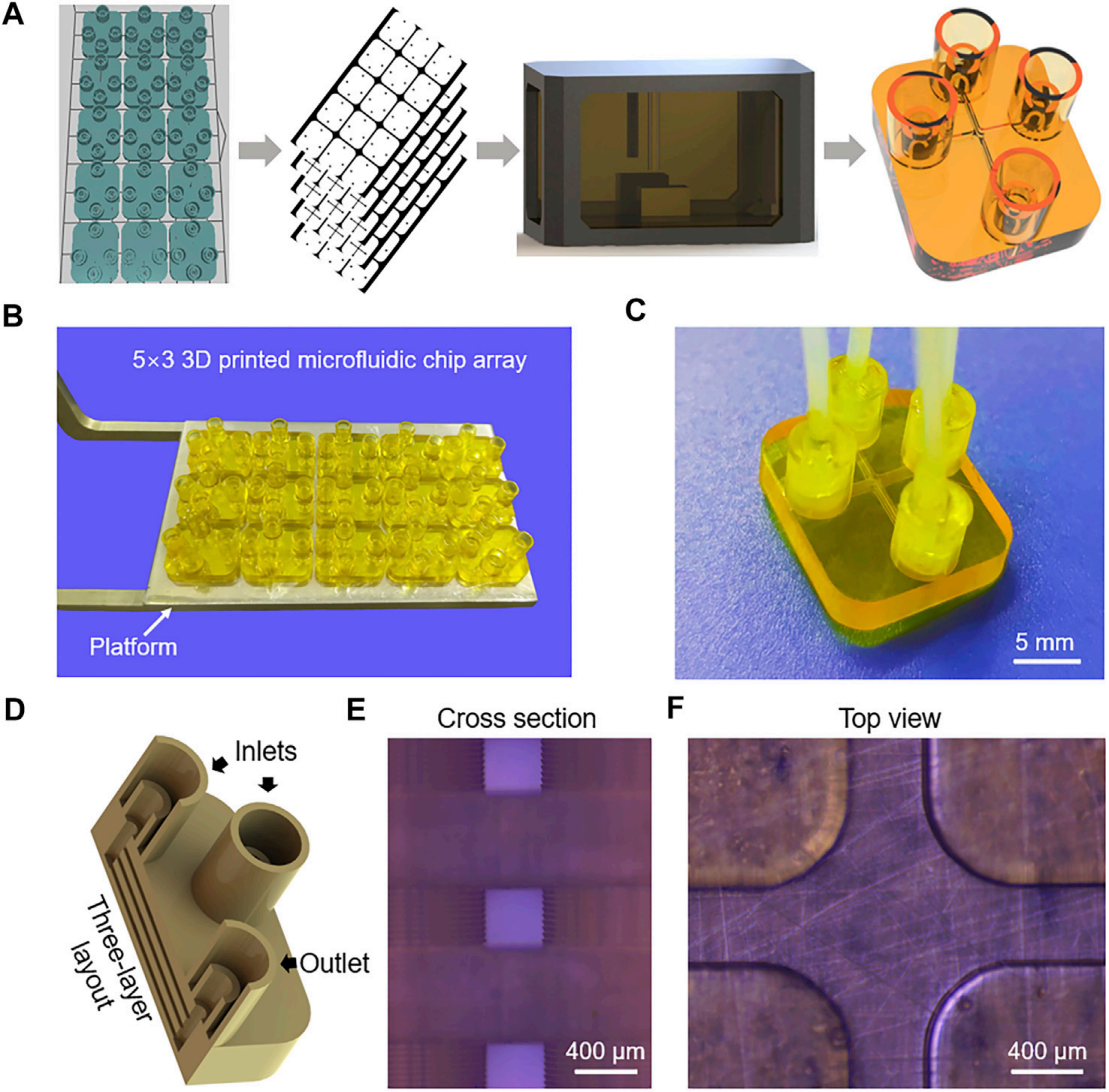
**(A)** Schematic diagram of the PμSL 3D printing process for fabricating multi-layer microfluidic chips. **(B)** Photograph of the 5 × 3 3D printed microfluidic chip array. **(C)** Photograph of the microfluidic chip connected with tubes. **(D)** The internal structure of the microfluidic chip. **(E)** Micrograph cross-sectional image showing the three-layer layout of the microchannels. **(F)** Micrograph image of the top view microchannel.

As shown in Figure 1A, a modeling software (Solidworks, Dassault Systemes, United States) was used to create the 3D model of the microfluidic chip with an STL format. Then, a slicing software (3D slicer; Boston Micro Fabrication, China) was used to slice the 3D model with a given thickness. A high-resolution PμSL 3D printer (microArch™ S140; Boston Micro Fabrication, China) was used to fabricate the microfluidic chip by a layer-by-layer method. After printing, the microfluidic chip was immersed in ethanol for ultrasonic cleaning 5 min for removing the uncured resin. Subsequently, the microfluidic chip was treated by an ultraviolet (UV) lamp for 3 min for improving strength and rigidity.

### Preparation of Liposomes

1,2-Dipalmitoyl-sn-glycero-3-phosphocholine (DPPC) and cholesterol were obtained from Xi’an Ruixi Biological Technology Co., Ltd., China. Isopropyl alcohol (IPA) was purchased from Shanghai Macklin Biochemical Co., Ltd, China. Phosphate buffered saline (PBS) (0.1 M, pH 7.4) was obtained from Codow Chemical Co., Ltd, China. To ensure the complete solution of lipids, DPPC and cholesterol were dissolved in IPA with a molar mass ratio of 7:3 by vortexing for 3 min. The Luer syringes with different dimensions were purchased from Taobao.com. The specific FRR and TFR were achieved by regulating the injection velocity of the syringes mounted on a precision linear stage (PSA150-11-X; Zolix, China). The liposome formation was conducted using 3D printed three-layer microfluidic chips, and each two devices were used with a specific FRR, with liposomes collected from three different runs on each chip.

### Characterization and Measurements

The cross-sectional and top view of the three-layer microchannels were observed by using an optical microscope (BX53M; Olympus, Japan). Dynamic light scattering (DLS) was applied to measure the size and polydispersity index (PDI) of the prepared nanoliposomes *via* Zetasizer (Nano ZS ZEN3600; Malvern, United Kingdom). The Z-Average and PDI values are the mean of three measurements. The characterization of the morphology of liposomes was conducted by transmission electron microscopy (TEM) (Tecnai G2 F20 S-TWIN; FEI, United States). For TEM measurement, the sample was prepared by applying a 20 μl drop of the liposomes to a 200-mesh copper grid. After 30 min, a 20 μl drop of phosphotungstic acid negative staining solution (2%) was applied on the grid. Then, the sample was air-dried at room temperature following the removal of the excess stain. The IPA concentration distribution in microchannel was imaged with an optical microscope (Kingyuk, China). Methylene blue was dissolved in IPA with a concentration of 50 mM for capturing the IPA concentration easily.

### Numerical Simulation of Flows in Microchannels

Numerical simulations of the velocity, pressure, and IPA concentration of flows in microchannels were conducted with a 3D model using COMSOL Multiphysics 5.4 (COMSOL, Inc., United States), Turbulence module. To make a comparison of multiple mixing conditions, models with different FRRs and TFRs that applied in experiments were simulated. The model dimension was consistent with microchannel geometry, which enabled a better understanding of the turbulent mixing characteristics in microchannels.

## Results and Discussion

### 3D Printing of Three-Layer Microfluidic Chip

Figure 1A schematically illustrates the 3D printing of microfluidic chips. A 3D model of the microfluidic chip with an STL format was imported into a slicing software. The length, width and height of the microfluidic chip are 18, 15, and 4 mm, respectively. Four connectors with a maximum diameter of 5 mm and a height of 6 mm are integrated with the microfluidic chip. Since the 3D printed microfluidic chip is produced by a layer-by-layer method and the slice thickness affects the resolution of the device directly, so the model was sliced with a relatively small thickness of 40 μm. Then, a high-resolution PμSL 3D printer was applied to fabricate microfluidic chips. An UV-curable resin (GR, Boston Micro Fabrication, China) was used to obtain the required structure of 3D printed devices. Here, GR is chosen to fabricate microfluidic chips for three reasons. Firstly, GR has relatively low viscosity and can yield the highest printing resolution. Secondly, the elastic modulus of GR is about 3.8 GPa, roughly three orders of magnitude greater than PDMS (Fitzgerald et al., 2019), enabling a high injection pressure and an ultra-high volumetric throughput of nanoliposome, which will be discussed below. Thirdly, GR has a certain degree of transparency, so the microfluidic chip can be easily observed and detected. The 5 × 3 3D printed microfluidic chip array fabricated on a platform is presented in Figure 1B, indicating the capability to fabricate microfluidic chips on a large scale. The 3D printer can simultaneously fabricate fifteen or more microfluidic chips, which extremely improves the productivity and consistency of the microfluidic chips. The 3D printed device for liposome preparation has three inlets, that is, one inlet of lipids and two inlets of the buffer. As shown in Figure 1C, three inlets and one outlet were designed to match the diameter of tubes, providing a convenient way to connect chips to external devices. The integrated connectors and tubes were sealed with epoxy resin, avoiding leakages of the solution under a high-pressure condition. The internal structure of the microfluidic chip is shown in Figure 1D, all the inlets and outlet part have a cylindrical channel with a depth of 3.5 mm, and the entrances and exits of the three-layer microchannels are connected to the cylindrical channels. Three identical microchannels located at different heights have a rectangular cross section with 400 μm depth and 400 μm width.

The microchannels of microfluidic devices have cross-sectional dimensions, typically in the range of 5–500 μm (Patil and Jadhav, 2014). Due to the sub-millimeter geometry of the microchannels, microfluidics generally only requires a droplet of samples to achieve an efficient analysis. However, differing from the conventional micro total analysis systems (μTAS) (Vilkner et al., 2004), the liposome formation microfluidic chips are more like a tool of production, which is necessary to ensure the repeatability and consistency of liposome size distribution from batch to batch. At present, it is still a challenge to realize a high volumetric throughput liposome preparation *via* microfluidic devices. Of note, 3D printing provides an alternative solution to the difficulties of fabricating microchannels located at different heights, enabling a higher volumetric flow rate in a single device. As is shown in Figure 1E, the three-layer microchannels were obtained by using the PμSL 3D printing method. The three-layer layout of microchannels can increase the volumetric flow rate without increasing the dimension of channels. Moreover, the flow distribution in three-layer microchannels is approximately equal and the stacked microchannels have the same microfluidic mixing conditions. In terms of the layers of microchannels, there is a trade-off between printing complexity and TFR. Although TFR can be further increased by introducing more layers of microchannels, it also means a longer printing time and a greater possibility of clogging issues. The minimum lateral resolution of the PμSL 3D printer is 10 μm, so the fabricated geometry of microchannels was highly consistent with the designed parameters (Figure 1F).

### Liposome Size Distribution Under Multiple Conditions

**FIGURE 2:**
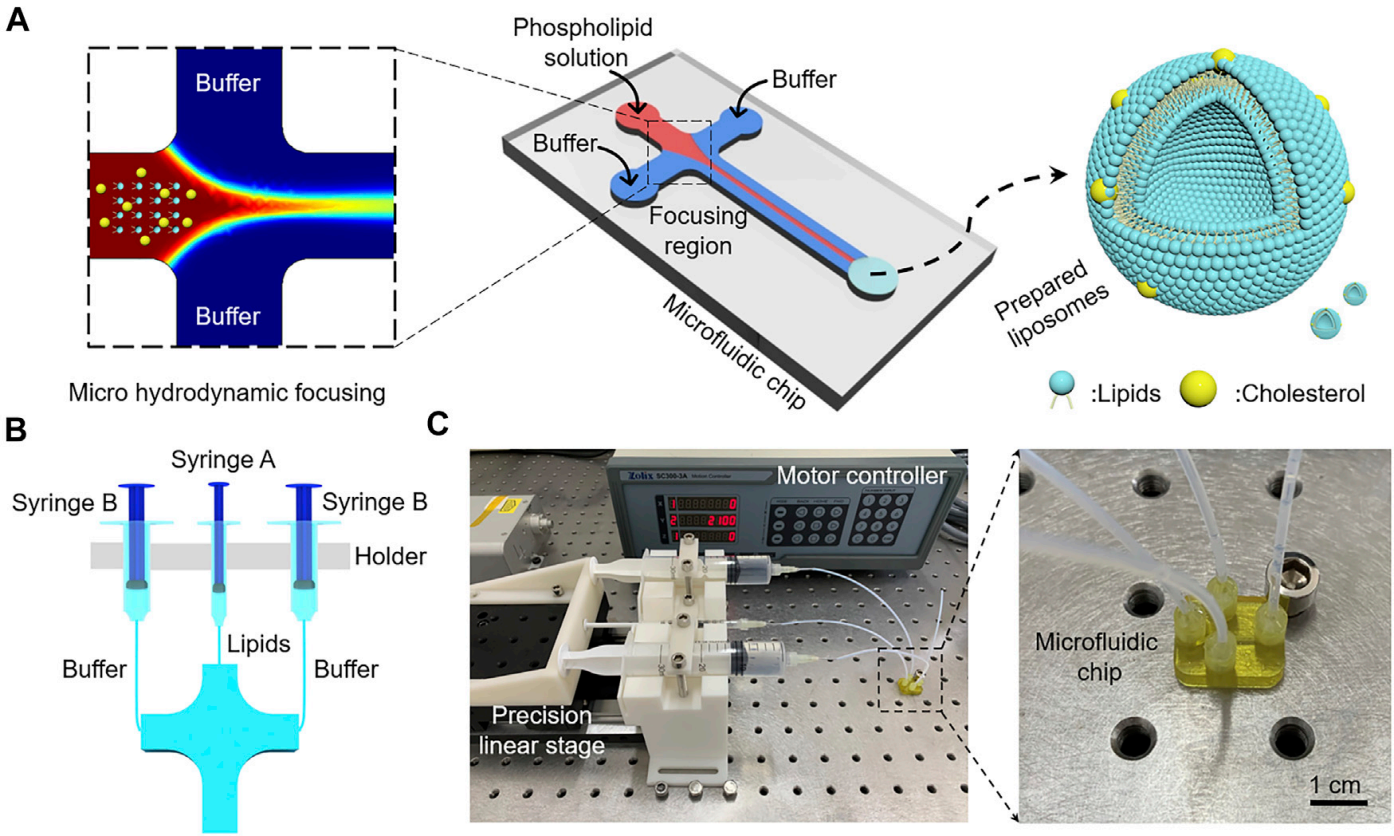
**(A)** Working mechanism of microfluidic-mediated liposome formation. **(B)** Schematic of syringes with different dimensions for injecting lipids and buffer in microchannels. **(C)** The process of microfluidic-mediated liposome formation using the 3D printed microfluidic chip.

Microfluidic-mediated liposome formation is a novel method to produce small size liposomes. Compared with the ether or ethanol injection method, the microfluidic method can accurately control the mixing condition of the solutions in microchannels. The working mechanism of the microfluidic method is depicted in Figure 2A, an organic solution containing phospholipids flows in the central channel and the aqueous buffer enters the vertical channel upwards and downwards. The concentration of organic solvent reduces in the mixing channel with the convective-diffusive mixing between the organic solution and aqueous buffer. Then, phospholipids are transferred to the aqueous buffer and spontaneously form phospholipid bilayers, eventually become liposomes.

Jahn et al. reported that microfluidic-mediated liposomes size distributions relate to FRR, TFR, device scaling, and so on (Jahn et al., 2010). As shown in Figure 2B, three syringes are used to inject lipids solution and buffer into microchannels. If syringe A and syringe B have the same injection speed, FRR is only relative to the inner diameters of syringes. Thus, FRR can be adjusted by using a range of syringes with different dimensions. Also, TFR can be easily changed by regulating the injection velocity of the syringe. The inner diameters of 1, 5, 10, 20, and 30 ml syringes in our work are 4.5, 12, 14.8, 19.8 and 22.2 mm, respectively. Table 1 shows a wide variety of calculated FRRs and TFRs in our work.

**TABLE 1:** A wide variety of calculated FRRs and TFRs in our work.

Syringe A (ml)	Syringe B (ml)	Velocity (mm s^−1^)	FRR	TFR (ml min^−1^)
1	5	5, 7.5, 10	14.2	72.6, 108.9, 145.2
1	10	5, 7.5, 10	21.6	108.0, 162.0, 216.0
1	20	5, 7.5, 10	38.7	189.5, 284.3, 379.0
1	30	5, 7.5, 10	48.7	237.0, 355.5, 474.0

The process of microfluidic-mediated liposome formation is shown in Figure 2C, a microfluidic chip with three-layer 400 × 400 μm rectangular microchannels was connected to three Luer lock syringes mounted on a precision linear stage. Three PEEK tubes with an internal diameter of 1 mm were used to connect the syringes and inlets. DPPC and cholesterol with a molar mass ratio of 7:3 were dissolved in IPA with a lipid concentration of 10 mM. To explore the highest volumetric throughput, a syringe of 1 ml was used to inject lipids solution into the central channel and two syringes of 30 ml were used to inject PBS buffer (0.1 M, pH 7.4) into the side channels, the selected velocity of the linear stage was 10 mm s^−1^, the calculated TFR was as high as 474 ml min^−1^ (Supplementary Movie S1), which is greatly higher than those in the previous references (Table 2) (Jahn et al., 2010; Hood et al., 2014; Carugo et al., 2016; Chen et al., 2019; Bishop et al., 2015).

**TABLE 2:** Comparison of TFRs in the reported references.

Reference	Depth, width of channel (μm)	FRR	TFR (ml min^−1^)
Our work	400, 400	48.7	474
Jahn et al. (2010)	120, 65	48	0.117
Hood et al. (2014)	112, 30	20	0.112
Carugo et al. (2016)	1000, 1000	100	18
Chen et al. (2019)	200, 8000	30	30
Bishop et al. (2015)	800, 800	-	2

The 3D printed microfluidic chip is formed integrally and is different from traditional chip consisting of microchannel layer and substrate, so the 3D printed chip can withstand a larger injection pressure than the traditional chip. As a result, the TFR of our 3D printed chip is greatly higher than the reported chips. Besides, the connectors and tubes were sealed with epoxy resin and no leakage was observed with the highest TFR, showing a stable world-to-chip interface under a high-pressure condition.

**FIGURE 3:**
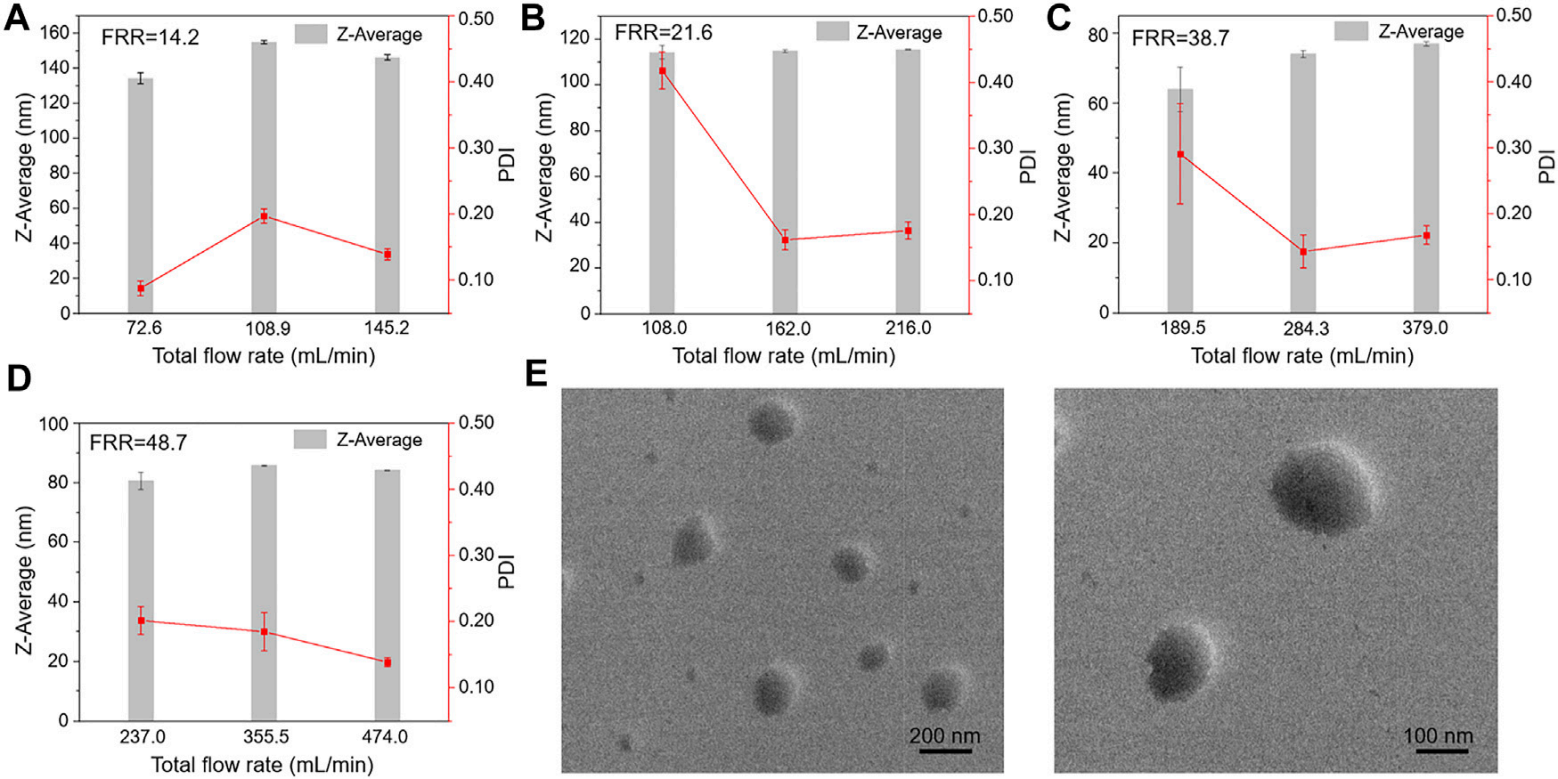
The Z-Average values of liposomes prepared at increasing TFRs for selected FRR. **(A)** FRR was 14.2. **(B)** FRR was 21.6. **(C)** FRR was 38.7. **(D)** FRR was 48.7. **(E)** TEM images of liposomes prepared at the highest TFR condition.

The diameter of liposomes directly influences the performance of liposomes, such as encapsulation efficiency and permeability (Akbarzadeh et al., 2013). All the experimental parameters shown in Table 1 were examined to obtain different FRRs and TFRs, and the liposome size distribution under multiple conditions was evaluated. DLS was used to measure the Z-Average and PDI of liposomes prepared at varying conditions. The value of Z-Average represents the average diameters of liposomes and PDI serves as a quantitative metric of size uniformity. Figure 3 shows the liposome size distribution with different FRRs and TFRs. As shown in Figure 3A, the FRR of buffer to lipids was 14.2, the Z-Average values of the liposomes prepared at increasing TFRs were 134.33 ± 3.21, 155.20 ± 1.00 and 146.33 ± 1.53 nm, respectively. When the FRR of buffer to lipids was 21.6 (Figure 3B), the Z-Average values were 114.32 ± 2.96, 114.85 ± 0.55 and 115.49 ± 0.1 nm, respectively. When the FRR was raised to 38.7 (Figure 3C), with the increase of TFR, the Z-Average values were 64.01 ± 6.38, 74.13 ± 1.04 and 74.98 ± 0.60 nm, respectively. As shown in Figure 3D, the FRR was 48.7 and the Z-Average values were 80.62 ± 2.95, 85.74 ± 0.17 and 84.15 ± 0.19 nm, respectively. The DLS results demonstrate that the 3D printed microfluidic chip can produce the liposomes with a diameter below 100 nm by introducing a large FRR. Furthermore, the PDI value of liposomes is not regular with the change of experimental parameters (Figures 3A–D and Supplementary Table S1).

To further verify the formation of liposomes under an ultra-high volumetric throughput condition, the morphology of liposomes was further characterized by TEM. Figure 3E shows the TEM images of liposomes prepared by using the 3D printed microfluidic chip with a TFR of 474 ml min^−1^. TEM exhibited that the prepared liposomes had a uniform and spherical morphology (from 100 to 200 nm in diameter), indicating the realization of the ultra-high volumetric throughput liposome preparation *via* 3D print microfluidic chips. We also noticed that the diameters of liposomes measured by TEM are a little greater than DLS results, due to the difference in measurement characteristics of the two techniques.

**FIGURE 4:**
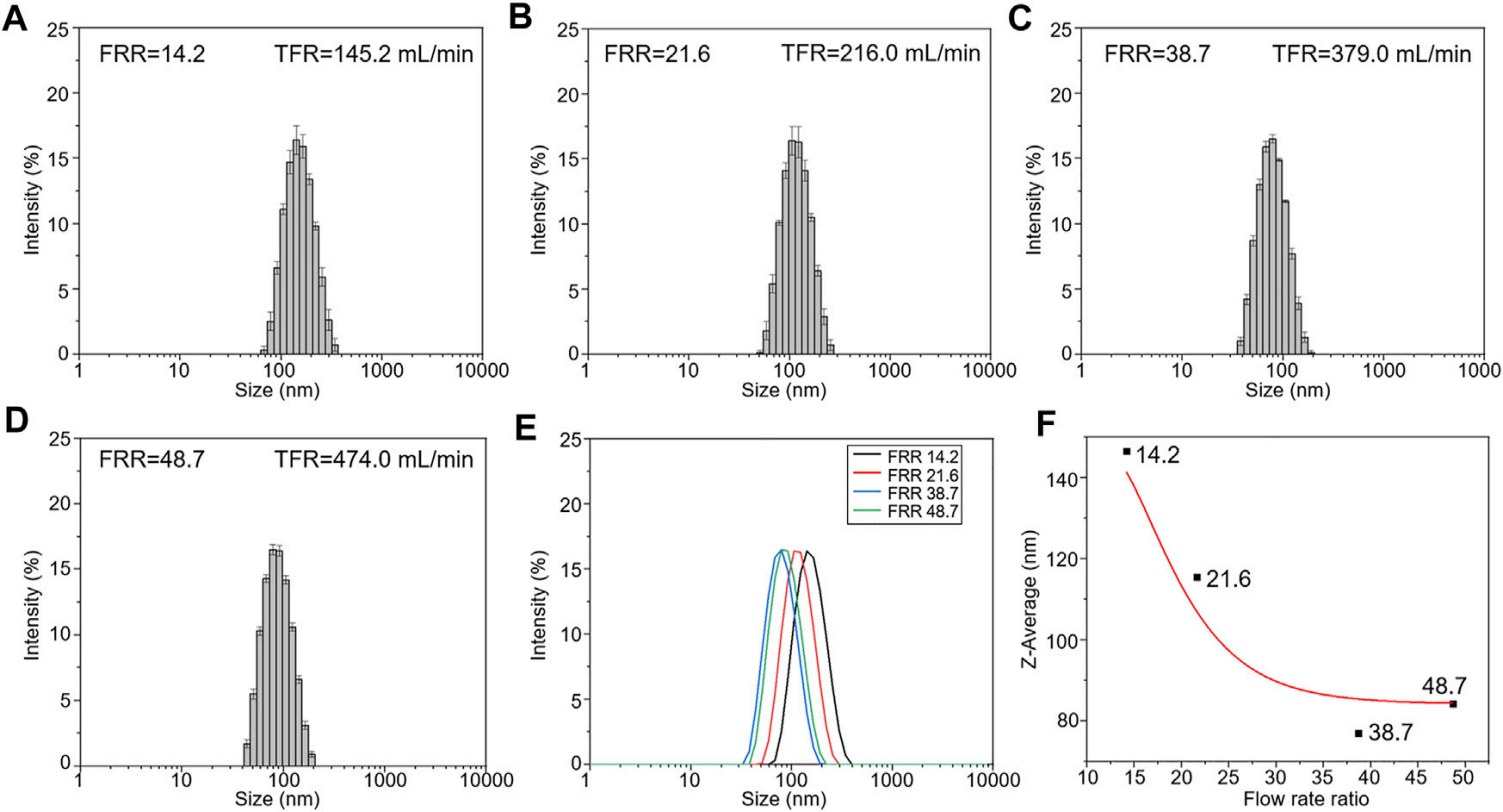
The size distribution of liposomes prepared at different FRRs. (The injection speed of syringes was 10 mm s^−1^). **(A)** FRR was 14.2. **(B)** FRR was 21.6. **(C)** FRR was 38.7. **(D)** FRR was 48.7. **(E)** The comparison of the liposome size distribution. **(F)** The fitting curve of influence of FRR on liposome diameters.

The comparison of the liposome size distribution with increasing FRRs is presented in Figure 4. Figures 4A–D show the size distribution of liposomes prepared at different FRRs. When FRR is 38.7, the liposomes have the smallest size, below 100 nm in diameter (Figures 4C,E). The fitting curve shows that the diameters of liposomes decrease with the increase of FRR within a certain range, and when FRR is above 35, the diameters remain almost unchanged (Figure 4F). The influence scope of FRR in our work is almost consistent with the previous literature (Jahn et al., 2010). The comparison of that under a lower volumetric throughput (the injection speed of syringes was 1 mm s^−1^) was also investigated. The results exhibited the same phenomenon in the influence of FRR on liposome diameters (Supplementary Figure S1). Meanwhile, compared to other 3D printed MHF devices, the size distribution of nanoliposomes reported here is similar with that reported in references (Chen et al., 2019; Tiboni et al., 2020). In addition, the diameter of liposomes in our work is slightly larger compared with that prepared by silicon-based microfluidic chips (Jahn et al., 2010), owing to the larger channel dimensions of 3D printed devices.

### The State of Flows in Microchannels

*Re* is a dimensionless parameter that defines the relative magnitude of the inertial force and the viscous force, which can be used to characterize the fluid flow. It can be described by the following equation:

Re=ρULμ=ULν

$$L=\frac{2ab}{\left(a+b\right)}$$

**FIGURE 5:**
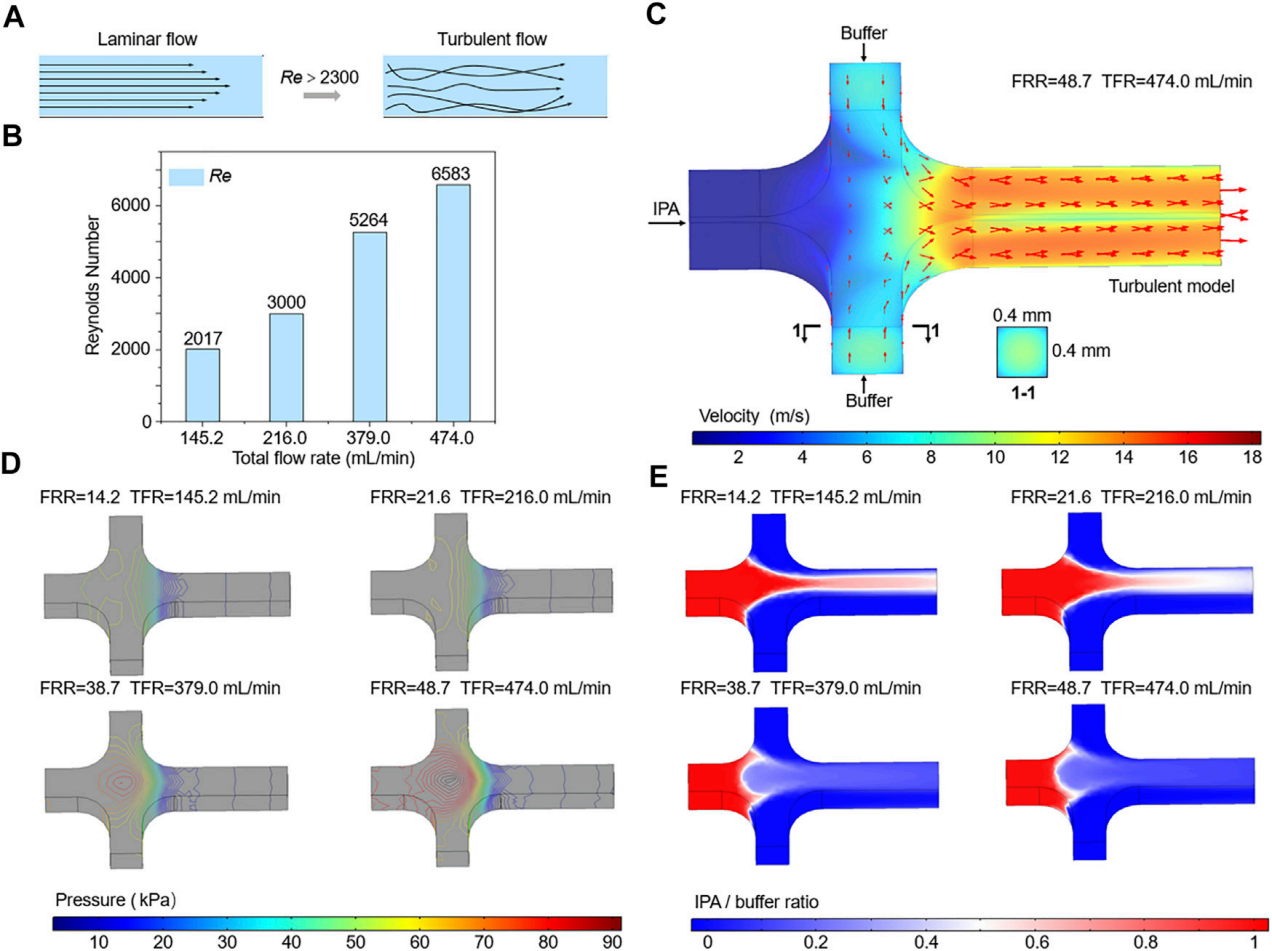
**(A)** Schematic of the transition from laminar to turbulence. **(B)** The calculated *Re* of the flows in the mixing channel. **(C**–**E)** Numerical simulations of the velocity, pressure, and IPA concentration of flows in microchannels.

Where *ρ* is the density of the flow, *U* is the flow velocity, *μ* is the dynamic viscosity of the fluid, and *υ* is the kinematic viscosity. *L* is the hydrodynamic diameter of the channel, *a* and *b* are the width and height of the channel, respectively. Generally, the streams that flow in microchannel have a low *Re* and the synthesis of liposomes depends on the diffusive mixing under laminar flow conditions. As TFR increases, *U* is dramatically increased and the value of *Re* is further raised. When the *Re* of the flow exceeds the critical value of 2300, it converts from laminar to turbulent flow (Figure 5A).

**FIGURE 6:**
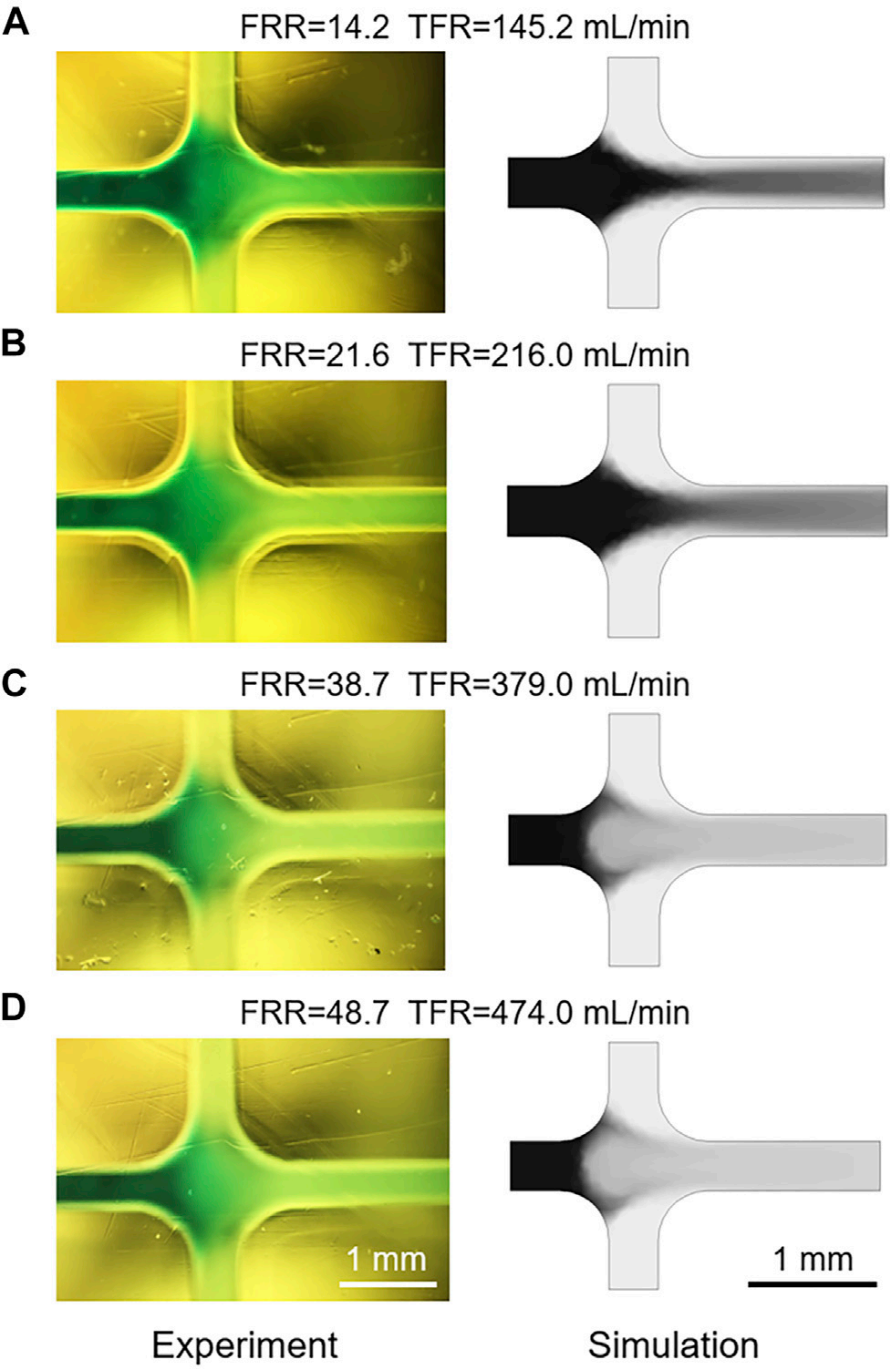
**(A**–**D)** The comparison of experimental and simulated IPA concentration distribution with different FRRs and TFRs.

As shown in Figure 5B, the *Re* values of flows in microchannels at different TFRs are compared. The calculated *Re* of the flow in the mixing channel with a TFR of 474 ml min^−1^ is 6583, which is far beyond the critical *Re* value. Therefore, flow state in the mixing channel can be divided into laminar and turbulence, according to the value of TFR. Due to the limitation of the low TFR and injection pressure, most of previous researches and applications only focus on the effect of laminar flow on liposome formation, while studies on the influence of turbulent flow are rarely reported. Differing from the negative prediction of turbulence, we found that flows in microchannels under turbulent flow conditions could generate small size liposomes, for example, the Z-Average of liposomes prepared with a TFR of 474 ml min^−1^ was 84.15 ± 0.19 nm (Figures 3D, 4D). To accurately demonstrate the turbulent mixing conditions in microchannels, a 3D turbulent model was established using COMSOL Multiphysics. Figure 5C demonstrates the model details and the velocity profile with the highest TFR of 474 ml min^−1^. With the increase of the FRR and TFR, the pressure in the focusing region is significantly enhanced (Figure 5D). Simulated IPA concentration profiles are shown in Figure 5E, the focused stream exists only at a relatively low FRR of 14.2 or 21.6. In contrast, when FRR is 38.7 or 48.7, there is an obvious phenomenon of turbulent mixing in focusing regions, which may attribute to the high injection pressure and velocity. To further validate the numerical simulation results, the concentration distribution of IPA containing methylene blue was observed by an optical microscope. As shown in Figure 6, the experimental and simulated results of IPA concentration distribution are in good agreement. The injection pressure is a crucial parameter that affects the liposome forming process. The preparation of liposomes under turbulent mixing conditions in our work is similar to that of crossflow injection method (Wagner et al., 2002), shear forces are more dominant than diffusion in liposome size reduction, which is suitable for producing liposomes with a quite small size. The experiments results demonstrate that the liposome size reduction is affected by the interaction between FRR and injection pressure, while no significant change is observed with the increase of TFR for the same FRR (Figures 3A–D).

## Conclusion

In summary, we fabricated an integrated multi-layer microfluidic chip for ultra-high volumetric throughput nanoliposome preparation using a PμSL 3D printer. The performance of the 3D printed multi-layer microfluidic chip for liposome preparation was evaluated. The microchannels of the 3D printed microfluidic chip adopt a three-layer layout, contributing to the highest TFR up to 474 ml min^−1^. The 3D printed microfluidic chip exhibited an effective liposome size control with a range of FRRs. The state of flows in the microchannel was estimated by comparing the *Re* values of flows with different TFRs, and the formation mechanism of liposomes under laminar or turbulent flow conditions was further explored. These results indicate that the 3D printed integrated microfluidic chip features ultra-high volumetric throughput and can produce nanoliposomes with controlled size efficiently, which enables further advances in molecular imaging and targeting drug delivery systems.

## Data Availability

The original contributions presented in the study are included in the article/Supplementary Material, further inquiries can be directed to the corresponding authors.
