# Supplementary material for: 3D Printed Integrated Multi-Layer Microfluidic Chips for Ultra-High Volumetric Throughput Nanoliposome Preparation
Source: Front Bioeng Biotechnol. 2021 Oct 11;9:773705. doi: 10.3389/fbioe.2021.773705 (PMC8542840; doi:10.3389/fbioe.2021.773705)
Supplement: Supplementary file 1 [file Table1.DOCX]

Supplementary Material

**Table S1.** The PDI values of the prepared liposomes with increasing FRRs and TFRs.

| FRR | PDI | | |
| --- | --- | --- | --- |
|  | 5 (mm s^-1^) | 7.5 (mm s^-1^) | 10 (mm s^-1^) |
| 14.2 | 0.088 ± 0.011 | 0.197 ± 0.011 | 0.139 ± 0.008 |
| 21.6 | 0.418 ± 0.028 | 0.162 ± 0.015 | 0.176 ± 0.013 |
| 38.7 | 0.291 ± 0.076 | 0.143 ± 0.025 | 0.168 ± 0.014 |
| 48.7 | 0.202 ± 0.021 | 0.185 ± 0.029 | 0.139 ± 0.007 |


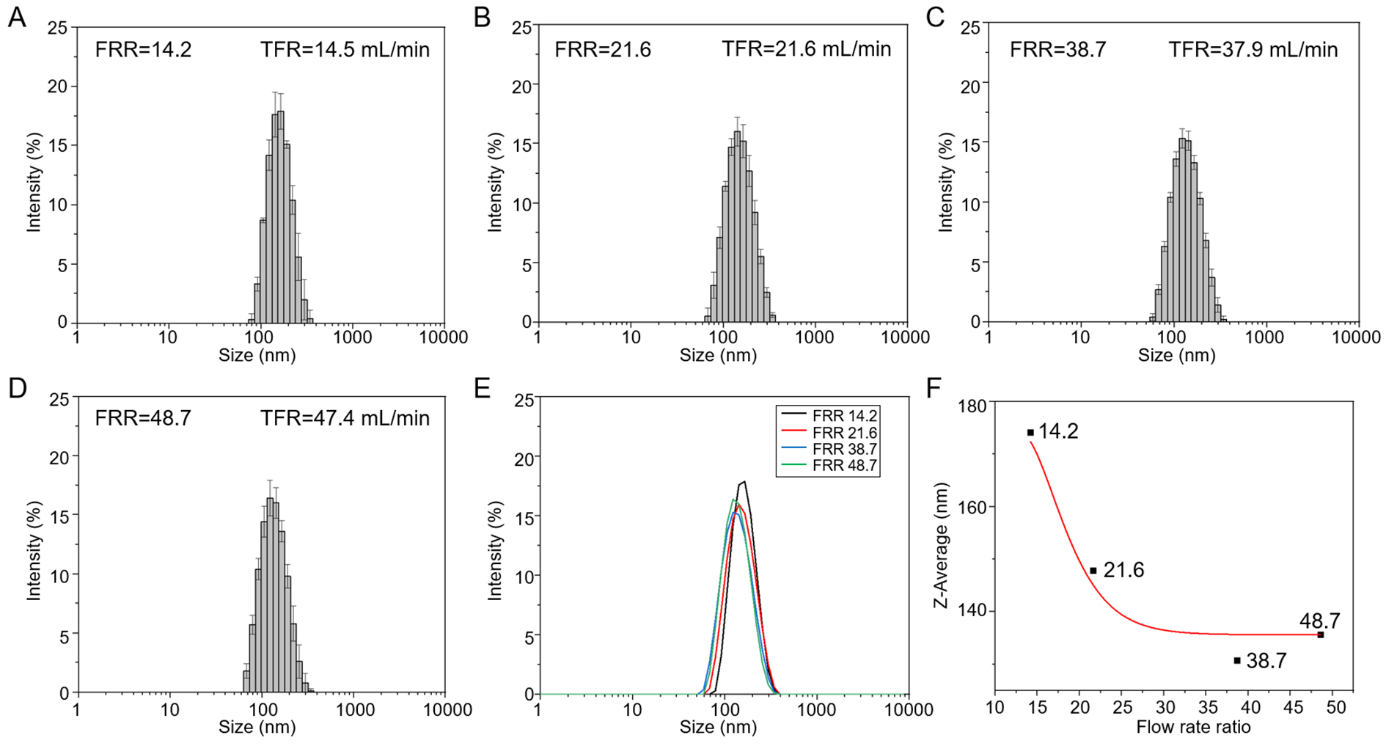


**Figure S1.** The size distribution of liposomes prepared at different FRRs under a lower volumetric throughput. (The injection speed of syringes was 1 mm s^-1^). **(A)** FRR was 14.2. **(B)** FRR was 21.6. **(C)** FRR was 38.7. **(D)** FRR was 48.7. **(E)** The comparison of the liposome size distribution. **(F)** The fitting curve of influence of FRR on liposome diameters.
